# Dr. Westervelt Banta

**Published:** 1901-07

**Authors:** 


Dr. Westervelt Banta, U. of N., '88, of Buffalo, died at his home at Perry and Hayward Streets, June 17, 1901, of cancer of the stomach. Dr. Banta's father was one of the original shipyard-men of Buffalo. He is survived by a widow, a mother, Sarah M. Banta, and two brothers, Dr. Rollin L. Banta and William S. Banta. The funeral was held from his late home and from St. Bridget's Church, June 20.

